# Differential autophagy power in the spinal cord and muscle of transgenic ALS mice

**DOI:** 10.3389/fncel.2013.00234

**Published:** 2013-11-26

**Authors:** Valeria Crippa, Alessandra Boncoraglio, Mariarita Galbiati, Tanya Aggarwal, Paola Rusmini, Elisa Giorgetti, Riccardo Cristofani, Serena Carra, Maria Pennuto, Angelo Poletti

**Affiliations:** ^1^Sezione di Biomedicina e Endocrinologia, Dipartimento di Scienze Farmacologiche e Biomolecolari, Centro di Eccellenza sulle Malattie Neurodegenerative, Università degli Studi di MilanoMilano, Italy; ^2^Centro InterUniversitario sulle Malattie Neurodegenerative, Università degli Studi di FirenzeMilano, Genova e Roma Tor Vergata, Italy; ^3^Department of Cell Biology, University Medical Center of GroningenGroningen, Netherlands; ^4^Department of Neuroscience and Brain Technologies, Istituto Italiano di TecnologiaGenova, Italy; ^5^Sezione di Fisiologia e Neuroscienze, Dipartimento di Scienze Biomediche, Metaboliche e Neuroscienze, Università degli Studi di Modena e Reggio EmiliaModena, Italy; ^6^Dulbecco Telethon Institute Laboratory of Neurodegenerative Diseases, Centre for Integrative Biology (CIBIO), University of TrentoItaly

**Keywords:** amyotrophic lateral sclerosis, motoneurons, autophagy, HSPB8, BAG3, BAG1, protein quality control

## Abstract

Amyotrophic lateral sclerosis (ALS) is a motoneuron disease characterized by misfolded proteins aggregation in affected motoneurons. In mutant SOD1 (mutSOD1) ALS models, aggregation correlates to impaired functions of proteasome and/or autophagy, both essential for the intracellular chaperone-mediated protein quality control (PQC), and to a reduced mutSOD1 clearance from motoneurons. Skeletal muscle cells are also sensitive to mutSOD1 toxicity, but no mutSOD1 aggregates are formed in these cells, that might better manage mutSOD1 than motoneurons. Thus, we analyzed in spinal cord and in muscle of transgenic (tg) G93A-SOD1 mice at presymptomatic (PS, 8 weeks) and symptomatic (S, 16 weeks) stages, and in age-matched control mice, whether mutSOD1 differentially modulates relevant PQC players, such as HSPB8, BAG3, and BAG1. Possible sex differences were also considered. No changes of HSPB8, BAG3, and BAG1 at PS stage (8 weeks) were seen in all tissues examined in tg G93A-SOD1 and control mice. At S stage (16 weeks), HSPB8 dramatically increased in skeletal muscle of tg G93A-SOD1 mice, while a minor increase occurred in spinal cord of male, but not female tg G93A-SOD1 mice. BAG3 expression increased both in muscle and spinal cord of tg G93A-SOD1 mice at S stage, BAG1 expression increased only in muscle of the same mice. Since, HSPB8-BAG3 complex assists mutSOD1 autophagic removal, we analyzed two well-known autophagic markers, LC3 and p62. Both LC3 and p62 mRNAs were significantly up-regulated in skeletal muscle of tg G93A-SOD1 mice at S stage (16 weeks). This suggests that mutSOD1 expression induces a robust autophagic response specifically in muscle. Together these results demonstrate that, in muscle mutSOD1-induced autophagic response is much higher than in spinal cord. In addition, if mutSOD1 exerts toxicity in muscle, this may not be mediated by misfolded proteins accumulation. It remains unclear whether in muscle mutSOD1 toxicity is related to aberrant autophagy activation.

## Introduction

Autophagy is a fundamental intracellular degradative pathway activated to respond to the accumulation of aberrantly folded (misfolded) proteins (Mizushima and Komatsu, 2011). Autophagy is required for the cellular protein quality control (PQC) system, which also includes molecular chaperones and the ubiquitin-proteasome degradative system (UPS) (Carra et al., 2012). These systems work together protecting cells particularly sensitive to misfolded protein toxicity, such as motoneurons (Rusmini et al., 2010; Bendotti et al., 2012; Carra et al., 2013). Motoneurons are major targets of toxicity in diseases linked to mutant proteins prone to misfold, such as in amyotrophic lateral sclerosis (ALS) (Pasinelli and Brown, 2006; Rusmini et al., 2010; Sau et al., 2011; Strong and Yang, 2011; Carra et al., 2012, 2013; Robberecht and Philips, 2013). Most ALS cases appear in sporadic (sALS) forms; only about 10–15% have familial (fALS) history, and are clinically indistinguishable from sALS. fALSs have been associated with mutations in different genes, such as the superoxide dismutase 1 (SOD1), TAR DNA-binding protein 43 (TDP-43), fused in sarcoma/translocated in liposarcoma protein (FUS/TLS), optineurin (Robberecht and Philips, 2013), or the C9ORF72 gene (Dejesus-Hernandez et al., 2011; Renton et al., 2011; Ash et al., 2013; Lashley et al., 2013; Mori et al., 2013). Notably, also the wild type (wt) forms of the mutated fALS proteins may show aberrant behavior in sALS (e.g., oxydized wtSOD1, cleaved C-terminus of wtTDP-43, etc.) (Neumann et al., 2006; Daoud et al., 2009; Bosco and Landers, 2010; Bosco et al., 2010), suggesting the existence of a common pathological mechanism. An explanation for this is that these proteins (either the modified wt or the mutant forms) have the propensity to misfold and aggregate forming insoluble inclusions that are a key neuropathological hallmark of ALS. Inclusions may alter several cellular functions, such as axonal transport, mitochondrial, and/or degradative activities, thereby leading or contributing to motoneuron death (Pasinelli and Brown, 2006; Cozzolino et al., 2008; Seetharaman et al., 2009).

In recent years, emerging evidences support the idea that also non-neuronal cells (e.g., surrounding astrocytes or Schwann cells, chemotactically attracted microglial cells, and target muscle cells) might contribute to disease onset and progression (Boillée et al., 2006), by making motoneuronal cells more sensitive to protein toxicity. For example, selective expression of mutant SOD1 (mutSOD1) in skeletal muscle induced atrophy associated with the loss of motoneurons in the anterior horn of the spinal cord (Dobrowolny et al., 2008a; Corti et al., 2009; Wong and Martin, 2010). However, mutSOD1 largely accumulates in spinal cord of transgenic mice expressing human G93A-SOD1 (Tg G93A-SOD1) (Cheroni et al., 2005, 2009; Basso et al., 2006, 2009; Bendotti et al., 2012), but not in skeletal muscle of the same mice at any stage of disease (Galbiati et al., 2012; Wei et al., 2012); thus, muscle cells better cope with misfolded mutSOD1 species than motoneuronal cells. This may be due to an higher muscular degradative capabilities compared to motoneurons (Onesto et al., 2011). In fact, in both cells, mutSOD1 clearance involves both UPS and autophagy, but both the proteasome and autophagy activities are higher in muscle cells than in motoneuronal cells.

The interplay between UPS and autophagy is finely regulated through a mechanism that involves the co-chaperones BAG1 and BAG3, which act as a switch between the two pathways (Luders et al., 2000; Carra et al., 2008a; Gamerdinger et al., 2009; Arndt et al., 2010; Zhang and Qian, 2011). BAG1 is expressed at relatively higher levels in young tissues, paralleled by higher UPS activity, while BAG3 is expressed at higher levels in aged tissues, characterized by higher autophagic activity (Gamerdinger et al., 2009), and the BAG3:BAG1 ratio determines the fate of client proteins to be degraded through either UPS or autophagy. Indeed, BAG1 routes substrates to UPS by interacting with a complex formed by the chaperone HSC70 and the ubiquitinating enzyme CHIP. UPS overwhelming or impairment, due to misfolded proteins, results in upregulation of BAG3 and its partner, the chaperone HSPB8 (a small heat shock protein involved in misfolded proteins recognition) (Wang et al., 2008; Du et al., 2009; Crippa et al., 2010b; Gentilella and Khalili, 2011; Carra et al., 2013). The increased BAG3:BAG1 ratio favors a stoichiometric HSC70/CHIP complex association to BAG3/HSPB8 complex, allowing the p62-mediated autophagic removal of CHIP-ubiquitinated substrates (Crippa et al., 2010b; Gamerdinger et al., 2011; Zhang and Qian, 2011; Carra et al., 2012, 2013). Interestingly, we found that mutSOD1 blocks the autophagic flux in motoneurons, but not in muscle cells (Onesto et al., 2011). This autophagic flux blockage can be counteracted by HSPB8, which participates together with BAG3/HSC70/CHIP in the autophagic clearance of several different misfolded proteins (polyQ containing proteins, mutSOD1, a truncated form of TDP-43) (Carra et al., 2005, 2008a,b, 2013; Crippa et al., 2010b). In addition, HSPB8 induces translation attenuation via phosphorylation of eIF2alpha (Carra et al., 2009), thereby decreasing the amount of aggregation-prone proteins to levels manageable by PQC.

In this study, we analyzed whether spinal cord, which comprises the two main cell targets of SOD1 toxicity, namely motoneuronal and astroglial cells, and skeletal muscle, differentially activate the HSPB8 mediated PQC system in response to mutSOD1 in tg G93A-SOD1 mice. The results demonstrate that the autophagic response of muscle tissue to the mutSOD1 expression is much higher than that found in the spinal cord of the same mice, suggesting that if mutSOD1 exerts toxicity in muscle, this may not be mediated by misfolded protein accumulation.

## Materials and methods

### Animals

All the procedures involving animals and their care were carried out following the institutional guidelines and in accordance with national (D.L. no. 116, G.U. suppl. 40, Feb. 18, 1992), and international laws and policies (EEC Council Directives 86/609, OJ L 358, 1 DEC.12, 1987), and were approved by the Italian Institute of Technology Animal Care Committee. All the animals were kept under controlled temperature and humidity conditions with standardized dark/light cycles of 12 h each. Food (standard pellets) and water were supplied *ad libitum*. BL6JL Tg(SOD1)2Gur/J (Stock number 002297, Charles River, Wilmington, MA, USA) male mice or BL6JL-Tg(SOD1^*^G93A)2Gur/J (Stock number 002726, Charles River) male mice were crossed with wt female mice purchased (Stock number 100012, Charles River) or obtained in house by crossing C57Bl/6J female and SJL male mice. All the experiments were performed in mice coming from the F1 generation of the cross described above. Non-transgenic littermates were used as controls (NTg). Mice were genotyped by PCR on tail DNA as previously described (Gurney et al., 1994), using REDExtract-N-Amp Tissue PCR kit (Sigma-Aldrich, St. Louis, MO, USA). To evaluate disease stages, starting from the 8th week of age and twice a week, mice were tested for deficit by rotarod, and hanging wire by the same operator as previously described (Palazzolo et al., 2009). Body weight loss was also monitored. Disease onset was set as the time at which the mouse permanently starts to lose body weight. Four mice per group were anesthetized with isoflurane and sacrificed at 8 or 16 weeks of age, corresponding to presymptomatic (PS) or symptomatic (S) stage of disease. Quadriceps muscles and spinal cord were rapidly collected after the sacrifice, snap frozen in liquid nitrogen, and conserved at −80°C until RNA and protein extraction.

### RNA extraction

Total RNA from frozen spinal cords or muscles was extracted using the standard TRI Reagent protocol based on the method developed by Chomczynski and Sacchi (Chomczynski and Sacchi, 1987). RNA was subsequently extracted in accordance to manufacturer's protocol (Sigma-Aldrich). The precipitated RNA was dissolved in RNase-free water. Total RNA (1 μ g) was treated for 15 min at room temperature with 1 U of DNaseI (Sigma-Aldrich). Samples were reverse-transcribed using the High-Capacity cDNA Reverse Transcription Kit (Life Technologies Corporation, Carlsbad, CA, USA) according to the manufacturer's instructions, in a 20 μ L volume. Primers for selected genes were designed via the Primer Express software (Life Technologies Corporation) and purchased from MWG Biotech (Ebersberg, Germany). Primer sequences were as follows: mouse HSPB8: 5′-ATA CGT GGA AGT TTC AGG CA -3′ (forward), 5′- TCC TTT GAC CTA ACG CAA CC -3′ (reverse); mouse BAG3: 5′- ATG GAC CTG AGC GAT CTC A -3′ (forward), 5′- CAC GGG GAT GGG GAT GTA -3′ (reverse); mouse BAG1: 5′- GAA ACA CCG TTG TCA GCA CT -3′ (forward), 5′- GCT CCA CTG TGT CAC ACT C -3′ (reverse); mouse MAP-LC3b: 5′- CGT CCT GGA CAA GAC CA -3′ (forward), 5′- CCA TTC ACC AGG AGG AA -3′ (reverse); mouse p62: 5′- AGG GAA CAC AGC AAG CT -3′ (forward), 5′- GCC AAA GTG TCC ATG TTT CA -3′ (reverse); mouse GAPDH: 5′-CCA GAA CAT CAT CCC TGC AT-3′ (forward), 5′-CAG TGA GCT TCC CGT TCA-3′ (reverse). The evaluated efficiency of each set of primers was close to 100% for both target and reference gene. Real-time PCR was performed using the CFX 96 Real Time System (Bio-Rad Laboratories, Hercules, CA, USA), in a 10 μ L total volume, using the iTaq SYBR Green Supermix (BioRad), and with 500 nmol primers. PCR cycling conditions were as follows: 94°C for 10 min, 40 cycles at 94°C for 15 s, and 60°C for 1 min. Melting curve analysis was performed at the end of each PCR assay to control specificity. Data was expressed as Ct values and used for the relative quantification of targets with the ΔΔCt calculation to give N-fold changes in gene expression. Values were normalized to those of GAPDH. To exclude potential bias due to averaging data transformed through the equation 2^−ΔΔCt^, all statistics were performed with ΔCt values. Each experiment was carried out with four independent samples, and each sample was run in duplicate wells.

The BAG3:BAG1 relative ratio was obtained considering the group of 8 weeks-old NTg female mice as control for the basal physiological condition of the PQC system. In particular, for each sample a single BAG3:BAG1 ratio was determined [measuring the (BAG3 2^−ΔΔCt^/BAG1 2^−ΔΔCt^)_sample_] and the mean BAG3:BAG1 ratio of control was subtracted [(BAG3 2^−ΔΔCt^/BAG1 2^−ΔΔCt^)_control_]. Finally, the resulting values were normalized by the mean BAG3:BAG1 ratio of the control 8 weeks-old NTg female mice.

### Western blotting and filter retardation assay (WB and FRA)

Total proteins from frozen spinal cord or muscles were extracted in 1% SDS using the standard TRI Reagent protocol, in accordance with the manufacturer's protocol (Sigma-Aldrich). Protein concentration was determined with the bicinchoninic acid method (BCA assay, EuroClone, Pero, Milan, Italy). WB analysis was performed on 12% sodium dodecyl sulfate (SDS)-polyacrylamide gel electrophoresis loading 15 μ g of total proteins. Samples were then electrotransferred to Nitrocellulose membrane (Bio-Rad) using the Trans-Blot Turbo (Bio-Rad). The membranes were treated with a blocking solution containing 5% non-fat dried milk powder (EuroClone) in TBS-T for 1 h and then incubated with the primary antibodies: (1) home-made rabbit polyclonal anti-HSPB8 (kindly provided by Dr Jacques Landry, Quèbec, Canada, dilution 1:1000); (2) rabbit-polyclonal anti-BAG3 antibody (Abcam, Cambridge, UK; dilution 1:1000); (3) rabbit-polyclonal anti-BAG1 (Santa Cruz Biotechnology, Dallas, Texas, USA; dilution 1:1000); (4) rabbit polyclonal anti-LC3 (Sigma; dilution 1:1000); (5) rabbit polyclonal anti-SQSTM1/p62 (Abcam; dilution 1:1000); (6) rabbit-polyclonal anti-GAPDH (Santa Cruz Biotech; dilution 1:1000); (7) rabbit-polyclonal anti-SOD1 (SOD-100; StressMarq, Victoria, BC, Canada; dilution 1:1000). Immunoreactivity was detected using goat anti-rabbit (sc-2004, Santa Cruz Biotech, dilution 1:5000) secondary peroxidase-conjugated antibody. Immunoreactivity was then visualized using the enhanced chemiluminescence detection kit reagent (ECL prime Western Blotting Substrate, GE Healthcare, Maidstone, UK). The same membranes were subsequently processed with different antibodies to detect the levels of different proteins in the same samples loaded on the gel, after stripping for 10 min at room temperature (StripABlot, EuroClone). Each experiment was carried out twice, with four independent samples.

FRA was performed using a slot-blot apparatus (Bio-Rad). 3 μ g of total proteins were loaded on a 0.2 μm cellulose acetate membrane (Whatman, GE Healthcare), pre-incubated with 20% methanol, washed with water and then incubated with 1% SDS solution. After slight vacuum filtration, slot-blot membranes were probed as described for WB.

A ChemiDoc XRS System (Bio-Rad) was used for the image acquisition of WB and FRA.

### Statistical analysis

Statistical analysis was performed through two-tailed Student *t-test* for comparisons between 8 and 16 weeks mice of the same group (NTg/Tg wtSOD1/Tg G93A-SOD1) and two-way analysis of variance (ANOVA) for group comparisons, using the PRISM software (GraphPad, San Diego, CA, USA). Specific group pair(s) statistical difference was determined by the Bonferroni *post-hoc* test. Data were expressed as mean ± SD of four independent samples.

## Results

**Figure 1:**
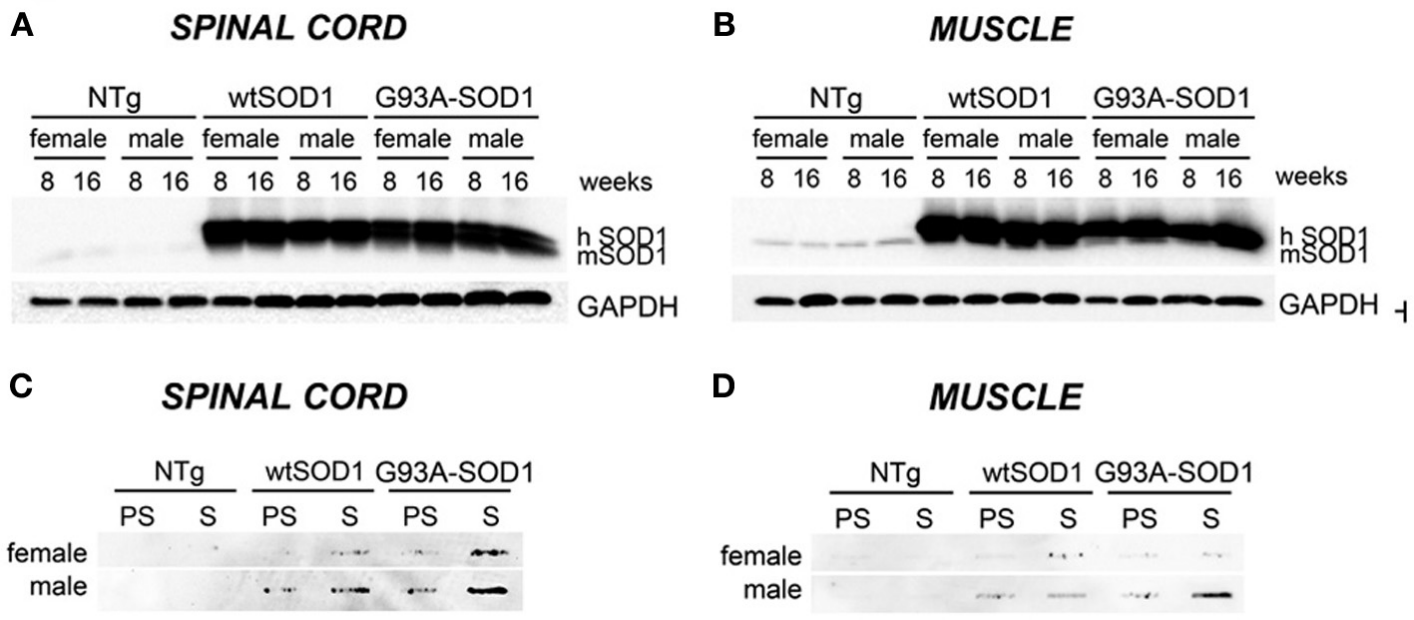
**Biochemical properties of wtSOD1 and mutSOD1 in spinal cord and muscle of ALS animal models**. Western Blot (WB) analysis and Filter Retardation Assay (FRA) on proteins extracted from whole spinal cord (**A**, WB; **C**, FRA) or quadriceps muscles (**B**, WB; **D**, FRA) of female and male non-transgenic (NTg) mice, of mice expressing the wild type human SOD1 transgene (wtSOD1), and of mice expressing the G93A mutant form of human SOD1 (G93A-SOD1), at 8 (corresponding to presymptomatic stage, PS, in Tg G93A-SOD1 mice) or 16 weeks (corresponding to symptomatic stage, S, in Tg G93A-SOD1 mice). GAPDH was used to normalize protein loading. hSOD1, transgenic human SOD1; mSOD1, endogenous murine SOD1.

Our study has been performed on 8 (presymptomatic stage, PS) and 16 weeks-old (symptomatic stage, S) Tg G93A-SOD1 mice, compared to age-matched non-transgenic (NTg) and transgenic human wild-type SOD1 (Tg wtSOD1) mice. In addition, since gender differences have been reported to affect the age of onset and disease progression both in ALS patients (Kurtzke, 1982; Giagheddu et al., 1983; Rudnicki, 1999; Manjaly et al., 2010; McCombe and Henderson, 2010; Lee et al., 2013a) and rodent models of ALS (Veldink et al., 2003; Suzuki et al., 2007), with male gender being more susceptible, we analyzed males and females separately. Based on our data on the protective role of HSPB8 in ALS, which is mediated by autophagy, we analyzed the expression of this small chaperone in the spinal cord and muscle of Tg G93A-SOD1 mice and control mice. We also analyzed the expression of the two co-chaperones BAG3 and BAG1, which may mediate the PQC activity by selecting the proper degradative system to be activated in cells in response to proteotoxicity. Finally, we analyzed two widely used autophagic markers, LC3 and p62. Using homogenates of spinal cord and skeletal muscle derived from Tg G93A-SOD1 mice and age-matched NTg or Tg wtSOD1 mice, we first compared the levels of wtSOD1 and mutSOD1 in both the soluble and insoluble fractions in 8 (corresponding to PS stage) and 16 weeks (corresponding to S stage) male and female mice. As shown in Figure 1, soluble monomeric wtSOD1 and mutSOD1 were analyzed by western blot (WB; **A**: spinal cord; **B**: muscle). In the spinal cord of Tg mice the levels of wtSOD1 and mutSOD1 proteins were similar, while in skeletal muscle mutSOD1 levels were apparently lower as compared to wtSOD1 and no gender differences were observed. Using motoneuronal and muscle ALS models as well as tg G93A-SOD1 mice, we recently demonstrated that mutSOD1 accumulated both in motoneuronal cells and in spinal cord of tg mice (already at PS, increasing at S stage), but did not accumulate in ALS muscle cells and ALS muscle tissues at any age tested (Onesto et al., 2011; Galbiati et al., 2012). These data were recently confirmed and extended by Wei and coll. (Wei et al., 2013) using a different strain of tg G93A-SOD1 mice. Thus, here we repeated these experiments to provide a general view of mutSOD1 behavior in our mice. A representative filter retardation assay (FRA; **C**: spinal cord; **D**: muscle) illustrating the corresponding insoluble fractions of mutSOD1 is reported. The FRA analysis confirmed the presence of relevant amounts of mutSOD1 insoluble species in samples of S stage tg G93A-SOD1 mice. In muscle tissue, no mutSOD1 insoluble species were present, with the exception of samples obtained from tg G93A-SOD1 male mice at S stage (16 weeks), suggesting that muscle tissue may better cope with the misfolded fraction of mutSOD1 as compared to cells in the spinal cord.

**Figure 2:**
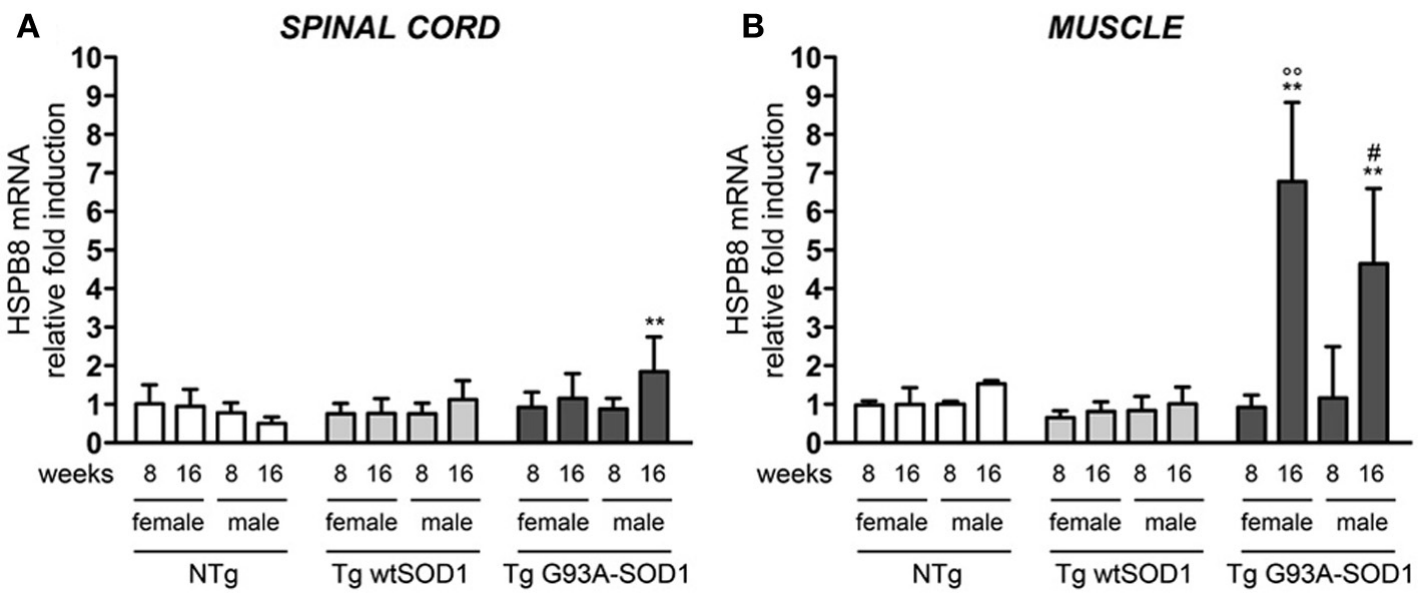
**Changes in HSPB8 levels in response to wt or mutSOD1 expression in spinal cord and muscle of ALS animal models**. RT-qPCRs were performed on total RNA extracted from whole spinal cord **(A)** or quadriceps muscles **(B)** of female and male non-transgenic (NTg) mice, of mice expressing the wild type human SOD1 transgene (Tg wtSOD1), and of mice expressing the G93A mutant form of human SOD1 (Tg G93A-SOD1), at 8 (corresponding to presymptomatic stage, PS, in Tg G93A-SOD1 mice) or 16 weeks (corresponding to symptomatic stage, S, in Tg G93A-SOD1 mice). Data have been normalized to the amount of GAPDH mRNA, expressed relative to the levels determined in age-matched PS female NTg mice, taken as internal reference, and expressed as fold changes. Data are mean ± SD of four independent replicates. **(A)** ^**^p < 0.01 vs. age- and sex-matched NTg mice. **(B)** ^**^p < 0.01 vs. age- and sex-matched NTg mice and Tg wt SOD1 mice; °°*p* < 0.01 vs. PS (8 weeks) female Tg G93A-SOD1; ^#^*p* < 0.05 vs. PS (8 weeks) male Tg G93A-SOD1.

Since we already showed that overexpression of HSPB8 prevents mutSOD1 intracellular accumulation by facilitating its clearance through autophagy (Crippa et al., 2010a,b), we analyzed HSPB8 mRNA levels in the spinal cord and skeletal muscle in response to wt or mutSOD1 expression. In spinal cord, we observed a significant increase of HSPB8 mRNA transcript levels in response to mutSOD1 expression in S stage (16 weeks) Tg G93A-SOD1 male mice as compared to age-matched NTg or Tg wtSOD1 mice (Figure 2A) (Crippa et al., 2010b). Interestingly, Tg G93A-SOD1 female mice did not show any significant increase of HSPB8 transcript level in response to mutSOD1xpression, suggesting the existence of gender differences in the PQC system activated by misfolded proteins in spinal cord. In male 16-week-old NTg mice, the expression level of HSPB8 tended to decrease, even if it did not become statistically significant. However, we already shown that HSPB8 expression significantly decreases when older NTg mice (age-matched with Tg G93A-SOD1 mice at end stage of disease) were analyzed (Crippa et al., 2010a,b). In contrast to what we found in spinal cord, in the skeletal muscles of S stage (16 weeks) Tg G93A-SOD1 mice we observed a dramatic increase of HSPB8 mRNA (up to 8-fold) (Figure 2B). Importantly, this HSPB8 mRNA upregulation occurred in both sexes. HSPB8 mRNA levels were unaffected in both sexes in NTg and Tg wtSOD1 mice at all ages considered.

**Figure 3:**
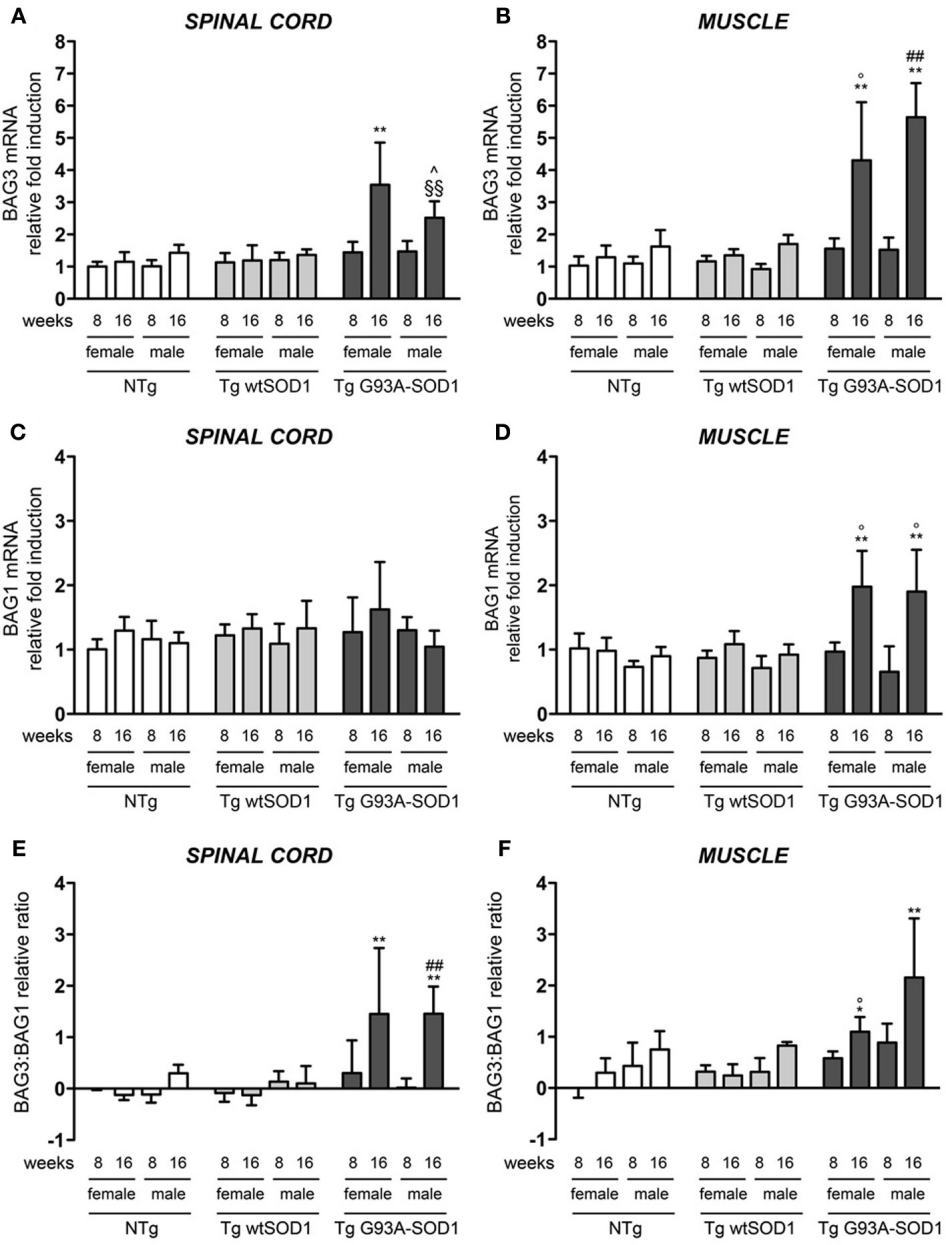
**BAG3 and BAG1 expression in spinal cord and muscle of ALS animal models**. RT-qPCRs were performed on total RNA extracted from whole spinal cord **(A,C)** or quadriceps muscles **(B,D)** of female and male non-transgenic (NTg) mice, of mice expressing the wild type human SOD1 transgene (Tg wtSOD1), and of mice expressing the G93A mutant form of human SOD1 (Tg G93A-SOD1), at 8 (corresponding to presymptomatic stage, PS, in Tg G93A-SOD1 mice) or 16 weeks (corresponding to symptomatic stage, S, in Tg G93A-SOD1 mice). Data have been normalized to the amount of GAPDH mRNA, expressed relative to the levels determined in age-matched PS female NTg mice, taken as internal reference, and expressed as fold changes. Data are mean ± SD of four independent replicates. **(A)** RT-qPCR on BAG3 mRNA expression levels in whole spinal cord. ^**^p < 0.01 vs. age- and sex-matched NTg and Tg wtSOD1 mice; ^∧^*p* < 0.05 vs. age- and sex-matched NTg mice; ^§§^*p* < 0.01 vs. age- and sex-matched Tg wtSOD1 mice. **(B)** RT-qPCR on BAG3 mRNA expression levels in quadriceps muscles. ^**^p < 0.01 vs. age- and sex-matched NTg and Tg wtSOD1 mice; °*p* < 0.05 vs. PS (8 weeks) female Tg G93A-SOD1; ^##^*p* < 0.01 vs. PS (8 weeks) male Tg G93A-SOD1. **(C)** RT-qPCR on BAG1 mRNA expression levels in whole spinal cord. **(D)** RT-qPCR on BAG1 mRNA expression levels in quadriceps muscles. ^**^p < 0.01 vs. age- and sex-matched NTg and Tg wtSOD1 mice; °*p* < 0.05 vs. sex-matched PS (8 weeks) Tg G93A-SOD1. **(E)** BAG3:BAG1 relative ratio of mRNA expression levels in whole spinal cord. Data represent variations of the relative levels of BAG3 and BAG1 normalized over the relative BAG3 and BAG1 levels of age-matched PS (8 weeks) female NTg mice (taken as internal reference, see Materials and Methods for details). ^**^p < 0.01 vs. age- and sex-matched NTg and Tg wtSOD1 mice; ^##^*p* < 0.01 vs. PS (8 weeks) male Tg G93A-SOD1. **(F)** BAG3:BAG1 relative ratio of mRNA expression levels in quadriceps muscles. Data have been calculated as in **(E)**. ^*^p < 0.05 and ^**^p < 0.01 vs. age- and sex-matched NTg and Tg wtSOD1 mice; °*p* < 0.05 vs. PS (8 weeks) male Tg G93A-SOD1.

The robust induction of HSPB8 expression in response to mutSOD1 in muscle might serve to initiate and/or facilitate the clearance of aberrant mutSOD1 species. This may avoid the aggregation of misfolded protein and the accumulation to levels that cannot be managed by the PQC system. Considering that mutSOD1 species are degraded by the saturable UPS, but it can be preferentially degraded by the highly efficient autophagic system in presence of high levels of HSPB8 (Crippa et al., 2010b) and that the pro-degradative activity of HSPB8 is mediated by its partner BAG3, we analyzed the expression levels of BAG3 in both spinal cord and muscle (Carra et al., 2008b; Carra, 2009). We found that in the spinal cord of both male and female mutSOD1 mice BAG3 expression remains unchanged at PS stage (8 weeks), but it was significantly increased by approximately 3-fold compared to NTg or Tg wtSOD1 mice at S stage (16 weeks, Figure 3A). Furthermore, the mutSOD1-induced appearance of symptoms correlated with a great response of the HSPB8-BAG3-mediated PQC system also in muscle (Figure 3B). Curiously, in female mice, BAG3 expression differed from that of HSPB8, since it was upregulated by mutSOD1 both in spinal cord and in muscle of tg G93A-SOD1 mice at S stage.

In light of our previous results showing that muscle cells possess a higher UPS activity with respect to motoneuronal cells (Onesto et al., 2011), we analyzed the expression of BAG1, which is involved in the re-routing of the misfolded proteins to proteasome-mediated substrates degradation. In spinal cord, BAG1 transcript levels were not affected by mutSOD1 expression (Figure 3C). On the contrary, BAG1 expression increased 2-folds in the skeletal muscle of S stage Tg G93A-SOD1 mice compared to all other groups analyzed (Figure 3D). This increase was similar in both sexes. These data suggest that proteasome-mediated substrate degradation is highly favored in muscle cells under proteotoxic conditions (e.g., misfolded mutSOD1 overexpression). We also estimated the ratio BAG3:BAG1 in each sample (Figures 3E,F) and found that this is always more elevated in muscle tissue, both in control and tg G93A-SOD1 mice, suggesting a predominance of the autophagic pathway, but it reaches higher levels at S stage of disease in response to mutSOD1. In the case of spinal cord, the BAG3:BAG1 ratio increases only at S stage (16 weeks) both in male and female tg G93A-SOD1 mice, suggesting that misfolded mutSOD1 could be re-routed to the autophagic system at this stage of disease (possibly not fully removed because of autophagic flux blockage).

**Figure 4:**
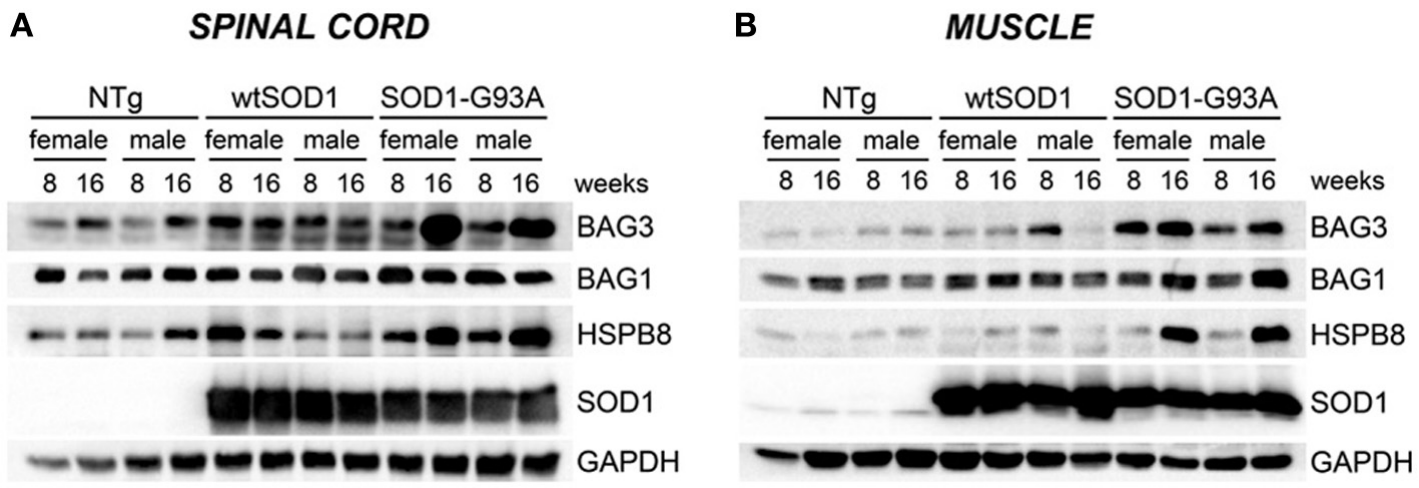
**HSPB8-BAG1-BAG3 protein levels in spinal cord and muscle of ALS animal models**. Western Blot (WB) analysis on proteins extracted from whole spinal cord **(A)** or quadriceps muscles **(B)** of female and male non-transgenic (NTg) mice, of mice expressing the wild type human SOD1 transgene (Tg wtSOD1), and of mice expressing the G93A mutant form of human SOD1 (Tg G93A-SOD1), at 8 (corresponding to presymptomatic stage, PS, in Tg G93A-SOD1 mice) or 16 weeks (corresponding to symptomatic stage, S, in Tg G93A-SOD1 mice). GAPDH was used to normalize protein loading.

Next, we asked whether the upregulation of the transcript levels of the genes described above results in an increase in protein expression. As shown in Figure 4, in the spinal cord **(A)**, HSPB8, BAG1, and BAG3 protein levels correlated with the data of gene transcription observed in the same mice. In skeletal muscle, we found that HSPB8, BAG3, and BAG1 were all up-regulated in Tg G93A-SOD1 mice at the S stage (16 weeks) of disease compared to Ntg and Tg wtSOD1 mice (Figure 4B). In the case of BAG3, the protein was up-regulated also at the PS stage (8 weeks). Little variation was observed in Tg G93A-SOD1 mice at S stage, while no major sex differences were noted. Also these data were perfectly in line and corroborate the mRNA expression analysis data reported above.

**Figure 5:**
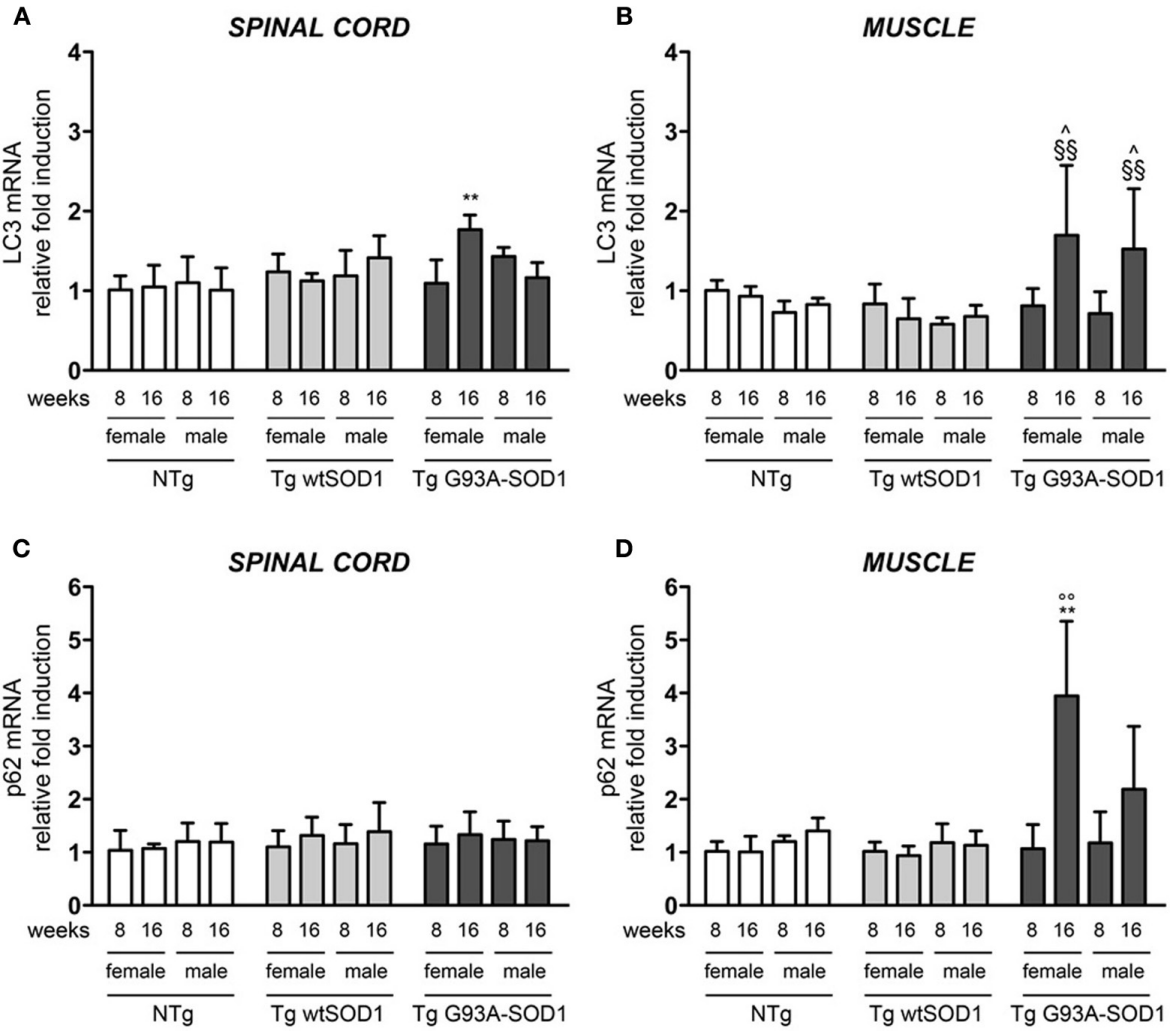
**LC3 and p62 expression in spinal cord and muscle of ALS animal models**. RT-qPCRs were performed on total RNA extracted from whole spinal cord **(A,C)** or quadriceps muscles **(B,D)** of female and male non-transgenic (NTg) mice, of mice expressing the wild type human SOD1 transgene (Tg wtSOD1), and of mice expressing the G93A mutant form of human SOD1 (Tg G93A-SOD1), at 8 (corresponding to presymptomatic stage, PS, in Tg G93A-SOD1 mice) or 16 weeks (corresponding to symptomatic stage, S, in Tg G93A-SOD1 mice). Data have been normalized to the amount of GAPDH mRNA, expressed relative to the levels determined in age-matched PS female NTg mice, taken as internal reference, and expressed as fold changes. Data are mean ± SD of four independent replicates. **(A)** RT-qPCR on LC3 mRNA expression levels in whole spinal cord. ^**^p < 0.01 vs. age- and sex-matched NTg and Tg wtSOD1 mice. **(B)** RT-qPCR on LC3 mRNA expression levels in quadriceps muscles. ^∧^*p* < 0.05 vs. age- and sex-matched NTg mice; ^§§^*p* < 0.01 vs. age- and sex-matched Tg wtSOD1 mice. **(C)** RT-qPCR on p62 mRNA expression levels in whole spinal cord. **(D)** RT-qPCR on p62 mRNA expression levels in quadriceps muscles. ^**^p < 0.01 vs. age- and sex-matched NTg and Tg wtSOD1 mice; °°*p* < 0.01 vs. PS female (8 weeks) Tg G93A-SOD1.

Since HSPB8 overexpression has beneficial effects in cell models of fALS, it is upregulated in human tissues from patients, and its mode of action requires the partner BAG3 and a functional autophagy system, we then analyzed the expression of two widely used markers of the autophagic flux: LC3 (which is overexpressed and associated with the autophagosome when autophagy is activated) and p62 (which is also upregulated during autophagy activation and is responsible for the insertion of ubiquitinated misfolded protein species into the autophagosomes). We found a moderate increase of LC3 mRNA selectively in the spinal cord of S stage (16 weeks) Tg G93A-SOD1 female mice (Figure 5A). In contrast, in skeletal muscle of Tg G93A-SOD1 mice (Figure 5B), LC3 expression was robustly increased at S stage both in male and female animals. p62 mRNA levels remained unchanged in spinal cord (Figure 5C), while they were increased in muscle of S stage female mice (Figure 5D). Notably, p62 levels increased also in Tg G93A-SOD1 male mice at S stage, even if this induction was not statistically significant.

**Figure 6:**
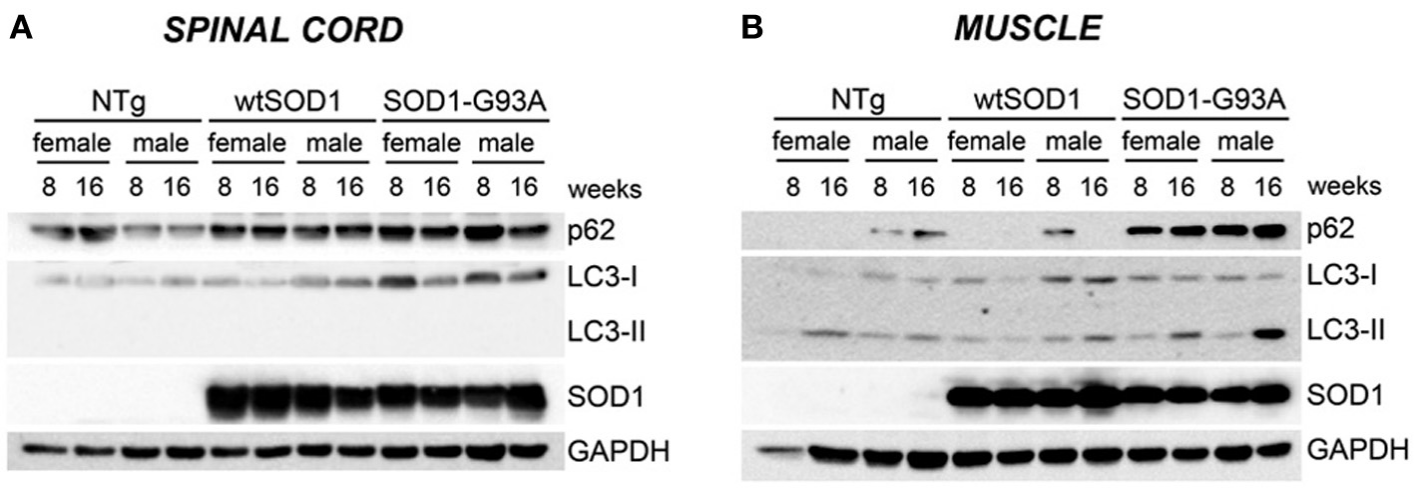
**Autophagic-markers (p62 and LC-3) protein levels in spinal cord and muscle of ALS animal models**. Western Blot (WB) analysis on proteins extracted from whole spinal cord **(A)** or quadriceps muscles **(B)** of female and male non-transgenic (NTg) mice, of mice expressing the wild type human SOD1 transgene (Tg wtSOD1), and of mice expressing the G93A mutant form of human SOD1 (Tg G93A-SOD1), at 8 (corresponding to presymptomatic stage, PS, in Tg G93A-SOD1 mice) or 16 weeks (corresponding to symptomatic stage, S, in Tg G93A-SOD1 mice). GAPDH was used to normalize protein loading.

Protein levels of LC3 and p62 as well as the conversion of free LC3-I to autophagosome-associated lipidated form of LC3-II were analyzed by WB (Figure 6). In spinal cord samples no major variations of p62 protein were observed, while the levels of LC3 protein were higher in Tg G93A-SOD1 mice (Figure 6A). Surprisingly, no conversion of LC3-I to LC3-II was observed, suggesting that the autophagic process is not massively activated in the spinal cord at these stages of disease. In skeletal muscle, p62 protein levels robustly increased in Tg G93A-SOD1 mice at both ages and in both sexes (Figure 6B). Moreover, at S stage (16 weeks), in skeletal muscle samples, the lipidated form of LC3 (LC3-II), which is the only one capable to associate with the newly formed autophagosomes, was increased in Tg G93A-SOD1 mice.

Collectively, these data strongly suggest that muscle, probably because of higher HSPB8, BAG1, and BAG3 expression, not only may better cope with misfolded mutant proteins, but also has a more intense autophagic response power compared to cells in the spinal cord.

## Discussion

In a previous study, we showed that spinal cord and muscle differently cope with mutSOD1 (Onesto et al., 2011). In fact, in Tg G93A-SOD1 mice mutSOD1 tends to accumulate in the spinal cord, while no accumulation of high molecular weight (HMW) species was found in muscles (Galbiati et al., 2012). Thus, the specific ability of spinal cord and skeletal muscle to handle misfolded protein aggregation and clearance (Cheroni et al., 2005, 2009; Basso et al., 2006, 2009; Bendotti et al., 2012; Galbiati et al., 2012; Wei et al., 2012) could be related to differences in their response to proteotoxicity, mediated by the PQC system. In this study we further investigated this aspect and found that mutSOD1 poorly accumulates in skeletal muscle, and only a mild increase of SDS-insoluble species of mutSOD1 is detectable in this tissue in Tg G93A-SOD1 male mice at the S stage (16 weeks), but not in female mice. This suggests that differences in the PQC system may also exist between the two sexes. Consequently, we then evaluated whether the expression pattern of specific factors of the PQC system shows a different expression profile in the spinal cord and muscle of a tg ALS mice model also considering possible gender differences.

To this purpose, here, we evaluated whether modifications occur at the initial stages of the PQC system, by focusing our attention on the HSPB8-BAG3 mediated pathway, involved in the autophagic process as well as on BAG1 pathway, involved in UPS mediated PQC. We then further investigated the autophagic process, since mutSOD1 induced alterations of UPS have already been analyzed by several investigators, reviewed in Bendotti et al. (2012), including us (Onesto et al., 2011). Autophagic response was analyzed using two well-known autophagic markers: LC3 and p62.

Our data clearly demonstrated that in both spinal cord and muscle HSPB8, BAG1, and BAG3 expression levels are not affected at early stage of disease (PS). The HSPB8-mediated PQC response was induced at the S stage of disease, and this activation was lower in spinal cord than muscle. Therefore, in spinal cord, the presence of misfolded mutSOD1 might not be sufficient to induce the activation of the PQC system *per se*, unless the disease is in an advanced phase, when motoneurons loss and/or alterations of muscle structures are present (Crippa et al., 2010b). Considering BAG3 and BAG1 expression, we found that their behavior was similar to that observed for HSPB8 at S stage (16 weeks). However, the BAG3 response appeared higher than that of BAG1, and BAG3 induction was also present in the spinal cord at S stage. Thus, cells in spinal cord have a lower capability than muscle cells to activate the HSPB8/BAG3- and BAG1-mediated PQC responses, and this might explain the larger accumulation of mutant misfolded proteins in this structure. It is of note that mutSOD1 expression results in the induction of both HSPB8 and BAG3 in the same structures, since HSPB8 activity is enhanced by BAG3 recruitment. Once the HSPB8-BAG3 interaction occurs, the complex recognizes the misfolded proteins and recruits the constitutively expressed chaperone HSC70 bound to the CHIP ubiquitinating enzyme (Arndt et al., 2010), allowing misfolded proteins removal by autophagy (Carra et al., 2008a,b, 2012, 2013; Arndt et al., 2010; Crippa et al., 2010b). In fact, CHIP ubiquitinates the target misfolded protein, which is then recognized by the protein p62 for its delivery to the nascent autophagosome (Arndt et al., 2010). Thus, HSPB8, BAG3, and the autophagy pathway may represent key players in the stress response elicited by mutSOD1 expression. Therefore, changes in their expression levels may correlate with the efficiency with which spinal cord and muscle cope with aggregate-prone mutSOD1.

On the other hand, the complex HSC70/CHIP can also associate to BAG1 to re-route substrates to UPS. When the proteasome component of UPS is overwhelmed or impaired by the misfolded proteins, HSPB8 and BAG3 are both highly induced. UPS inhibition is indeed a condition which occurs in motoneurons and muscle cells expressing mutSOD1 (Onesto et al., 2011), and the increase in the BAG3:BAG1 ratio allows BAG3 to more efficiently direct the HSC70/CHIP–associated substrates to autophagy for degradation (Zhang and Qian, 2011). Our data are in line with this working hypothesis, since in muscle we estimated that the BAG3:BAG1 ratio in tg G93A-SOD1 mice at S stage (16 weeks) is much higher than that observed in control mice. Conversely, in the spinal cord of tg G93A-SOD1 mice at S stage the BAG1 response is not detectable.

By following the auophagic response in muscle, our data here clearly show that at the S stage, muscle cells are still able to upregulate two essential players of the autophagic process, (a) p62, which recognizes the CHIP-ubiquitinated misfolded proteins in the HSPB8-BAG3-HSC70-CHIP complex, allowing their insertion into the autophagosome, and (b) LC3, which, after its activation to LC3, it is recognized by p62 and assists formation of autophagosomes. These steps are critical to initiate a correct autophagic flux required to clear the large excess of misfolded proteins from the cells during PQC response. This response does not efficiently take place in the spinal cord, since at S stage (16 weeks), misfolded mutSOD1 does not induce the overexpression of p62 and LC3, suggesting that the autophagic response in this structure is not as efficient as it is in muscle. In addition, in spinal cord very low basal levels of LC3-II form can be detected, suggesting that at these ages very few autophagosomes can be generated in response to mutSOD1. These data are consistent with previous reports obtained in the spinal cord of early symptomatic tg G93A-SOD1 mice, in which the LC3-I to LC3-II conversion, and thus, autophagy activation, occurs at very low levels, and it efficiently takes place only after the 17 week of age (Tian et al., 2011; Lee et al., 2013b). With disease progression, several autophagosomes appear in spinal cord motoneurons surviving at the end stage of disease (Li et al., 2008; Crippa et al., 2010b). This autophagic response can also be selectively activated in different cell types; for example LC3-I to LC3-II conversion can be visualized in cultured astrocytes in response to mutSOD1 (Kim et al., 2013).

With regards to the possible gender differences existing in the pathways here analyzed, we only found that, at S stage in tg G93A-SOD1 mice, the expression of p62 is significantly induced in skeletal muscle of female but not in that of male mice. These observations may help to explain why in skeletal muscle of tg G93A-SOD1 male mice mutSOD1 tends to accumulate in insoluble forms (see above). Interestingly, a mild increase of HSPB8 expression was present in spinal cord at S stage (16 weeks) of tg G93A-SOD1 male mice, but not in female mice. The molecular mechanism at the basis of this peculiar gender differences is unknown. Interestingly enough, there is a positive correlation between mutSOD1 accumulation and HSPB8 expression in the spinal cord of male tg G93A-SOD1 mice at S stage (16 weeks). This suggests that HSPB8 gene transcription increases in response to misfolded species accumulation. These data are in line with our previous results showing that an UPS impairment (which is know to result in a further mutSOD1 aggregation), lead to HSPB8 overexpression and stabilization (Crippa et al., 2010a,b; Bendotti et al., 2012; Carra et al., 2012, 2013). It remains to be determined why this HSPB8 overproduction fails to clear mutSOD1 insoluble species via autophagy (Crippa et al., 2010b; Rusmini et al., 2010).

By analysing the molecular and biochemical behavior of mutSOD1, we have previously elucidated several potential mechanisms of neurotoxicity associated with accumulation of misfolded proteins in motoneuronal cells (Sau et al., 2007, 2011; Crippa et al., 2010a,b; Onesto et al., 2011). For instance, the formation of HMW mutSOD1 species and aggregation correlated with an altered intracellular mutSOD1 distribution (Sau et al., 2007; Crippa et al., 2010b). In these conditions, mutSOD1 nuclear bioavailability was reduced in motoneuronal, but not in muscle cells (Sau et al., 2007; Crippa et al., 2010a,b; Onesto et al., 2011), an aberrant biochemical behavior that may result in a decreased nuclear protection from free radical species (Sau et al., 2007). In addition, by evaluating mitochondrial superoxide induced oxidation, using MitoSOX (a mitochondrial specific fluorogenic dye oxidized by superoxide) in motoneuronal NSC34 cell models of ALS, we found that mutSOD1 expressing motoneuronal cells are more sensitive to a superoxide-induced oxidative stress when compared to wtSOD1 expressing motoneuronal cells (Onesto et al., 2011). These mitochondrial alterations were not found in muscle C2C12 cells expressing either wt or mutSOD1 (Onesto et al., 2011). Therefore, mutSOD1 altered intracellular bioavailability, due to sequestration into aggregates, might result in a less efficient protection against free radical species damages in different subcellular compartments (Sau et al., 2007; Onesto et al., 2011).

Our results are in line with a recent report showing that protein misfolding, mitochondrial dysfunction and muscle loss are not directly dependent on soluble and aggregated mutSOD1 in skeletal muscle of ALS (Wei et al., 2012). Moreover, soluble mutSOD1 does not have direct effects on mitochondrial dysfunction as determined by quantifying the release of reactive oxygen species (ROS) in skeletal muscle mitochondria (Wei et al., 2012).

Interestingly, dysfunction of skeletal muscle and degeneration of neuromuscular junctions precede disease onset and motoneurons loss in tg ALS mice (Frey et al., 2000; Fischer et al., 2004). Notably, while the reduction of mutSOD1 expression in the skeletal muscle of tg ALS mice has no effect on disease progression (Miller et al., 2006), the selective expression of mutSOD1 in skeletal muscle induces atrophy and mitochondrial abnormalities in this tissue (Dobrowolny et al., 2008a; Corti et al., 2009; Wong and Martin, 2010). Moreover, mutSOD1-induced muscle damage leads to loss of mutSOD1-negative motoneurons in the anterior horn of the spinal cord, suggesting that mutSOD1 neurotoxicity can be exerted at different levels and its restricted expression in muscle can be sufficient to induce an ALS-like disease in mice (Dobrowolny et al., 2005, 2008a,b).

The data presented here clarify that mutSOD1 activates distinct pathogenetic pathways in muscle and motoneuron cells. In addition, the aggregation-independent mutSOD1 toxicity in muscle can be monitored at transcriptional levels. In fact, in our muscle cell models of ALS we observed specific alterations in the expression of typical genes controlling muscle pathways activated by nerve injury, or muscular atrophy [e.g., MyoD, myogenin (two myogenic regulatory factors), and TGFbeta1, which are markers for muscle fiber damage or atrophy] (Galbiati et al., 2012). Thus, muscle cells are able to better manage misfolded mutSOD1 species, even if misfolding is an intrinsic property of mutSOD1, and it does not depend on the cell environment (Dobson, 2003). It remains to be clarified whether the autophagic activation induced by mutSOD1 in muscle initially protective, when excessive, may become one of the toxic events mediating the deleterious activity associated with this mutant protein (Nassif and Hetz, 2011; Zhang et al., 2011). This hypothesis has already been proved in a ALS-related motoneuron disease, the Spinal and Bulbar Muscular Atrophy (SBMA) linked to an expansion of a polyQ stretch in the androgen receptor (ARpolyQ) (Poletti, 2004; Rusmini et al., 2013). Even in this motoneuron disease the mutant ARpolyQ affects both spinal cord and muscle, but it has been shown that an enhanced autophagy exacerbated skeletal muscle atrophy (Yu et al., 2011). Indeed, the genetic inhibition of autophagy in muscle, through haploinsufficiency for Beclin-1, a component of the autophagic initiation complex, mitigates skeletal muscle atrophy and prolongs survival of a tg SBMA mouse model (Yu et al., 2011).

Therefore, the toxicity exerted by mutant misfolded proteins in muscle cells is probably not related to the classical mechanism of intracellular protein aggregation, but other mechanisms of toxicity may be involved.

### Conflict of interest statement

Maria Pennuto and Angelo Poletti received support from Siena Biotech (Italy). The other authors declare that the research was conducted in the absence of any commercial or financial relationships that could be construed as a potential conflict of interest.
